# Use of lung ultrasonography in the detection of pneumothorax among medical students and emergency physician

**DOI:** 10.1186/cc10194

**Published:** 2011-06-22

**Authors:** UAP Flato, HP Guimarães, G Petisco, F Bezerra, AB Cavalcante, O Berwanguer

**Affiliations:** 1 Instituto de Ensino e Pesquisa do Hospital do Coração - Associação do Sanatório Sírio, São Paulo - SP, Brazil

## Introduction

The use of lung ultrasound in the detection of pneumothorax is becoming routine in emergency departments and intensive care units in the United States and Europe [1]. The interposition of the visceral and parietal pleura (pleural-lung interface) produces pulmonary artifacts easily visualized by ultrasound and described initially by Lichtenstein and Meziere [2]. In evaluating the lung for pneumothorax, the most important finding is the presence or absence of lung sliding. The presence of pleural sliding essentially rules out a pneumothorax in the analyzed region and the absence of lung sliding indicates a high suspicion of disease. Organizations such as the American College of Emergency Physicians (ACEP) have demonstrated the short learning curve and prompt application to clinical practice of this use of lung ultrasound. There is already evidence, both in Brazil and beyond, that knowledge retention based on an educational model using computer simulation would be particularly useful in training Brazilian physicians in lung ultrasound if it was proven to be effective.

## Objective

To evaluate the sensitivity and specificity of diagnosis of medical students compared with emergency physicians (experts) in identifying pneumothorax by lung ultrasound.

## Methods

Students of 3 years of medical graduation participating in the module Radiology Emergency Medicine (*n *= 40) and emergency physicians (*n *= 11) with training in emergency medicine and intensive care, called experts, were invited to participate. The study subjects were assessed for the correct diagnosis of 20 cases of pneumothorax after training through classroom teaching of lung ultrasound lasting 2 hours addressing the recognition of artifacts in the lung and identification of pneumothorax Lung Sliding Lines B. Prior to training, medical students and emergency physicians had no prior knowledge or practice in emergency ultrasonography. We used video-clips of 10 positive and 10 negative real cases of pneumothorax obtained by an experienced examiner in lung ultrasound. The comparison between the two groups was described by the mean and standard deviation of hits in each group and tested by the nonparametric Mann-Whitney test. The agreement between raters overall and in each group was estimated by the kappa correlation coefficient. The difference between the agreement observers in each group was tested by *Z *test for proportions.

## Results

Students and experts did not have statistically different test scores as shown in Table 1. There was a high degree of agreement between raters both overall and in each isolated group.

**Table 1:** 

Appraiser	Sensibility (%)	Specificity (%)	PPV (%)	VPN (%)	Accuracy (%)
All	87.8	92.0	91.6	88.3	89.9
Undergraduates	87.3	91.0	90.7	87.7	89.1
Experts	90.0	95.5	95.2	90.5	92.7

## Conclusion

Medical students and medical experts are able to accurately identify pneumothorax, despite an abbreviated training time with no previous knowledge of ultrasound lung. Therefore the use of a simulation model based on lung ultrasound videos can be implemented in a systematic way to help health professionals and medical students in their training.
